# Longitudinal Assessment of Oxidative Stress Biomarkers During Physiological Pregnancy and Their Relevance for Maternal Healthcare

**DOI:** 10.3390/healthcare14131878

**Published:** 2026-06-27

**Authors:** Martina Valachovičová, Csilla Mišľanová

**Affiliations:** Institute of Nutrition, Faculty of Nursing and Health Professional Studies, Slovak Medical University, 833 03 Bratislava, Slovakia; martina.valachovicova@szu.sk

**Keywords:** pregnancy, oxidative stress, antioxidants, lipid peroxidation, vitamins, longitudinal study

## Abstract

**Background/Objectives:** Pregnancy is characterized by profound metabolic and physiological adaptations that influence systemic redox balance. Longitudinal data assessing trimester-specific changes in antioxidant status and oxidative stress markers remain limited. **Methods:** In this longitudinal study, plasma levels of non-enzymatic antioxidants (vitamins A, C, E, and carotenoids) and oxidative stress markers (malondialdehyde, conjugated dienes, protein carbonyls, and DNA strand breaks) were analyzed in 31 healthy non-smoking pregnant women during the first, second, and third trimester. Environmental tobacco smoke exposure was evaluated using urinary cotinine. Statistical analyses were performed using Friedman repeated-measures tests followed by Dunn’s post hoc comparisons with Benjamini–Hochberg false discovery rate correction. **Results:** Vitamin A was the only antioxidant that consistently decreased across all trimesters and represented the only antioxidant marker showing a consistent decline. In contrast, α-tocopherol, γ-tocopherol, xanthophylls, and lycopene increased significantly during gestation, whereas vitamin C remained relatively stable. Markers of oxidative damage, including malondialdehyde, conjugated dienes, protein carbonyls, and DNA strand breaks, showed significant trimester-dependent increases. Total antioxidant capacity (FRAP) remained unchanged throughout pregnancy. **Conclusions:** Physiological pregnancy is characterized by coordinated, marker-specific adaptations in systemic redox balance. Vitamin A was the only antioxidant showing a consistent decline across gestation, whereas several lipid-soluble antioxidants increased and total antioxidant capacity remained stable. These findings indicate that pregnancy is associated with increased oxidative activity accompanied by preservation of systemic antioxidant capacity rather than global antioxidant depletion.

## 1. Introduction

Pregnancy represents a unique physiological state characterized by profound metabolic, hormonal, and immunological adaptations necessary to support fetal growth and development. Among these changes, modulation of oxidative balance plays an important role. Oxidative stress arises when the production of reactive oxygen species (ROS) and reactive nitrogen species (RNS) exceeds the capacity of antioxidant defense systems, potentially resulting in oxidative damage to lipids, proteins, and nucleic acids [1,2,3].

Under physiological conditions, moderate oxidative stress participates in normal cellular signaling and placental development. However, excessive oxidative stress has been associated with several pregnancy complications, including preeclampsia, gestational diabetes mellitus, intrauterine growth restriction, and preterm birth [4,5,6,7,8,9,10,11].

Intrauterine Growth Restriction (IUGR) can increase the risk of neonatal complications and mortality. Damage caused during intrauterine development can cause permanent anatomical and functional changes in the fetus, which adversely affect postnatal development and increase morbidity and mortality. The consequences of placental injury can persist into adulthood and are associated with a higher incidence of type 2 diabetes, ischemic heart disease, stroke, and metabolic syndrome [12,13].

The placenta is considered a major source of reactive oxygen species during pregnancy due to its high metabolic activity and mitochondrial respiration [3].

The human organism possesses a complex antioxidant defense system that includes both enzymatic and non-enzymatic components. Together, these systems act synergistically to neutralize excessive ROS, repair oxidative damage, and modulate signaling pathways that are critical for fetal development. Enzymatic antioxidants include superoxide dismutase, catalase, and glutathione peroxidase, which neutralize reactive oxygen species and prevent oxidative damage. Non-enzymatic antioxidants include vitamins A, C, and E, carotenoids, and other dietary antioxidants that contribute to maintaining redox homeostasis [14,15,16].

Several studies have demonstrated that the balance between oxidative stress and antioxidant defense changes throughout pregnancy as maternal metabolism increases and fetal development progresses [17,18,19]. For example, increasing oxidative activity during late pregnancy has been linked to enhanced mitochondrial function and increased oxygen consumption in placental tissues [17]. At the same time, maternal antioxidant systems may adapt to counteract elevated oxidative stress.

Vitamins with antioxidant properties, such as vitamin C, vitamin E, and carotenoids, play an important protective role during pregnancy by scavenging free radicals and preventing lipid peroxidation in biological membranes [20,21,22]. Vitamin A is also essential for embryonic development and cellular differentiation, although its plasma concentration may decrease during pregnancy due to hemodilution and increased maternal–fetal transfer [23].

Despite growing interest in oxidative stress during pregnancy, previous studies have reported heterogeneous and sometimes inconsistent findings regarding antioxidant status and oxidative stress markers, likely due to differences in study populations, study design, analytical methods, and selected biomarkers [1,2,6,8,15,24]. In particular, most available data are derived from cross-sectional or case–control studies, while longitudinal investigations assessing simultaneous changes in both antioxidant defenses and oxidative stress markers throughout uncomplicated pregnancy remain limited [3,15,24,25].

Therefore, the aim of the present longitudinal study was to evaluate changes in antioxidant defense and oxidative stress markers across the three trimesters of pregnancy in a cohort of healthy non-smoking women. By assessing multiple biomarkers of oxidative damage (such as malondialdehyde, conjugated diens, protein carbonyls, DNA strand breaks in lymphocytes) together with key antioxidant vitamins (vitamins A, C, α- and γ-tcopherol, β-carotene, xantophyll and lycopene) this study aims to provide a more comprehensive understanding of oxidative balance during physiological pregnancy.

Importantly, understanding these physiological changes is essential not only from a mechanistic perspective but also for their potential application in clinical practice, particularly in improving prenatal monitoring and preventive healthcare strategies.

## 2. Materials and Methods

### 2.1. Study Design

This longitudinal observational study included healthy pregnant women recruited during routine prenatal care visits. Only non-smoking women with uncomplicated singleton pregnancies were included to minimize potential confounding effects on oxidative stress parameters [26,27]. Participants were followed throughout pregnancy, and biological samples were collected longitudinally from the same participants during the first (10–12 weeks), second (20–24 weeks), and third trimester (34–36 weeks) of pregnancy (Table 1).

Inclusion criteria included healthy non-smoking women with uncomplicated singleton pregnancies. Exclusion criteria included pre-existing hypertension, diabetes mellitus, gestational diabetes, chronic inflammatory diseases, pregnancy complications, and the use of medications known to affect oxidative stress parameters (e.g., systemic corticosteroids, immunosuppressive agents, or long-term anti-inflammatory therapy).

Detailed dietary intake and adherence to prenatal vitamin supplementation were not quantitatively assessed.

Complete longitudinal data across all three trimesters were available for all 31 participants.

### 2.2. Sampling

Venous blood samples were collected from the antecubital vein after an overnight fast. Plasma and serum were separated by centrifugation and stored at −80 °C until analysis. Urine samples were collected concurrently with blood sampling for the assessment of environmental tobacco smoke exposure. Peripheral blood lymphocytes were isolated for the evaluation of DNA strand breaks. Table 2 lists the analyzed markers and the sample types in which they were analyzed.

### 2.3. Measurements

All high-performance liquid chromatography (HPLC) analyses were performed using the HP 1200 LC system (Agilent Technologies, Waldbronn, Germany) equipped with a quaternary pump with an online vacuum degasser, an autosampler, a thermostatted column compartment with Peltier cooling elements, a diode array (Agilent G1314D) or a fluorescence (Agilent G1321A) detector, and an electrically controlled internal six-port column-switching valve.

Validated HPLC methods were used to determine plasma levels of malondialdehyde (MDA) [28]; concentrations of plasma vitamin C [29]; α-tocopherol, γ-tocopherol and carotenoids-β-carotene, retinol, xanthophyll, and lycopene [30,31]. Lipid peroxidation was further assessed by conjugated dienes (CDs) [32], protein oxidation by protein carbonyl (PC) levels [33], and DNA damage in isolated lymphocytes using established protocols described in previous studies [34,35,36]. Total antioxidant capacity was assessed using the ferric reducing antioxidant power (FRAP) assay according to the method of Benzie and Strain [37]. All analytical procedures were performed according to validated laboratory methods commonly applied in oxidative stress research in human populations [38]. Internal quality control procedures and calibration standards were used throughout all analyses to ensure accuracy, precision, and reproducibility. Detailed methodological descriptions are provided in the Supplementary Materials.

### 2.4. Statistical Analysis

The study was designed as a descriptive longitudinal pilot study. Statistical analyses were performed using IBM SPSS Statistics version 28.0 (IBM Corp., Armonk, NY, USA). Data are presented as mean ± standard deviation (SD). Baseline characteristics are additionally described using descriptive statistics including maternal age and pre-pregnancy body mass index (BMI).

Normality of data distribution was assessed using the Shapiro–Wilk test. Due to the non-normal distribution of several variables and the fully repeated-measures design with complete data across all time points, non-parametric methods were applied.

Changes in biomarker levels across the three trimesters were evaluated using the Friedman test for repeated measures, followed by Dunn’s post hoc pairwise comparisons. To control for multiple testing across a large panel of biomarkers, *p*-values were adjusted using the Benjamini–Hochberg false discovery rate (FDR) procedure. Adjusted *p*-values (q-values) were reported, with q < 0.05 considered statistically significant. All tests were two-sided.

Given the complete-case longitudinal design (no missing data across the three trimesters), a non-parametric repeated-measures approach was considered appropriate and robust for the study structure. Although linear mixed-effects models (LMMs) are commonly used in longitudinal data analysis, the present dataset was balanced and complete for all participants at all time points, reducing the necessity for model-based imputation or random-effects modeling. Therefore, a Friedman repeated-measures approach was considered statistically appropriate for the balanced longitudinal dataset.

Additionally, given the pilot nature of the study and the large number of biomarkers evaluated, emphasis was placed on controlling the false discovery rate rather than on individual hypothesis testing.

The study was designed as a longitudinal descriptive pilot study.

## 3. Results

### 3.1. Markers of Oxidative Status

#### 3.1.1. Serum/Urine Antioxidants

Global differences across trimesters were assessed using the Friedman test, followed by Dunn’s post hoc comparisons with Benjamini–Hochberg FDR correction. All reported *p*-values refer to adjusted q-values unless otherwise stated.

Vitamin A concentrations showed a significant decrease during pregnancy, with lower levels observed in the third trimester compared with the first trimester (q = 0.013), while no significant differences were detected between adjacent trimesters. Among carotenoids, xanthophyll concentrations increased significantly from the first to the second trimester (q = 0.001) and from the first to the third trimester (q < 0.001). Similarly, lycopene concentrations were significantly higher in the second (q = 0.004) and third trimesters (q = 0.002) compared with the first trimester, with no significant difference between the second and third trimesters.

β-carotene concentrations showed an increasing tendency across pregnancy; however, none of the pairwise comparisons remained statistically significant (all q ≥ 0.067). α-tocopherol concentrations increased significantly between the first and second trimester (q < 0.001) and remained elevated in the third trimester compared with the first trimester (q < 0.001), with no difference between the second and third trimesters. A comparable pattern was observed for total vitamin E, with significant increases between the first and second trimester (q < 0.001) and between the first and third trimester (q < 0.001).

Vitamin C concentrations did not show consistent trimester-related differences after multiple testing correction, although a modest decrease was observed between the second and third trimesters (q = 0.033). The Vitamin C/Vitamin E ratio differed significantly between the first and second trimester (q < 0.001) as well as between the first and third trimester (q = 0.003).

#### 3.1.2. Markers of Oxidative Damage

MDA concentrations increased progressively, with significant differences observed between all trimester comparisons (all q ≤ 0.033).

Conjugated dienes were significantly higher in the third trimester compared with both the first and second trimesters (both q < 0.001), whereas no significant difference was observed between the first and second trimesters.

Protein carbonyl concentrations followed a similar pattern, with significantly higher levels in the third trimester compared with both earlier trimesters (both q < 0.001), while levels in the first and second trimesters did not differ significantly.

DNA strand breaks increased significantly between the first and third trimesters (q = 0.010), whereas differences between adjacent trimesters were not statistically significant after correction.

Total antioxidant capacity (FRAP) did not differ significantly across pregnancy (all q > 0.05).

**Figure 1:**
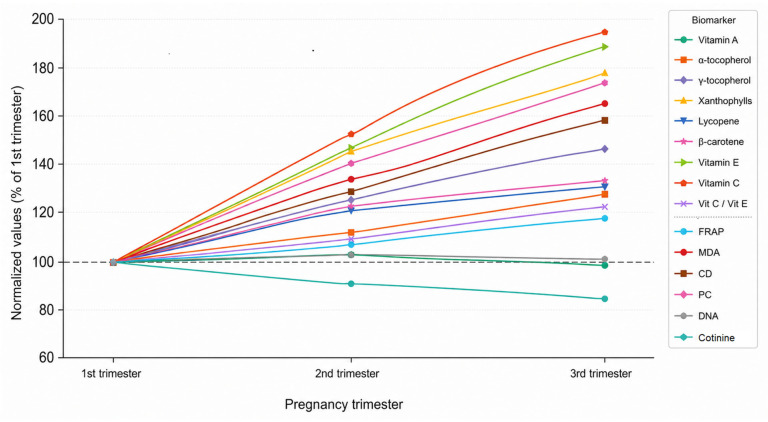
Longitudinal trajectories of antioxidant and oxidative stress biomarkers and cotinine across pregnancy.

**Figure 2:**
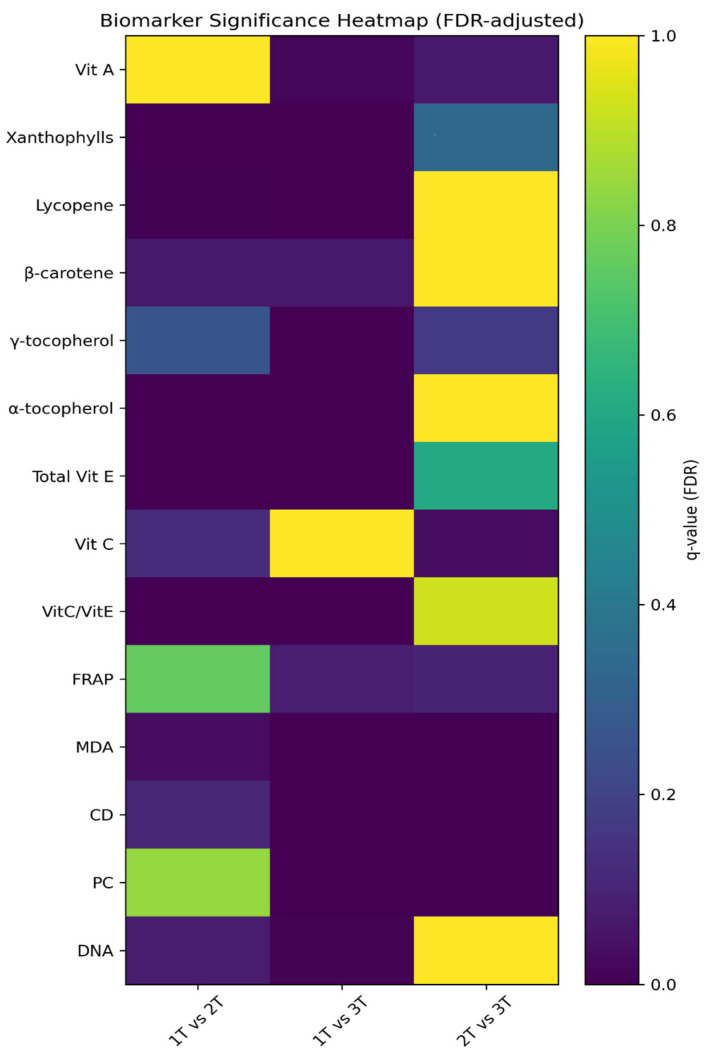
Heatmap of biomarker significance across pregnancy trimesters. Values represent FDR-adjusted *p*-values (q-values) obtained from Dunn’s post hoc test following Friedman analysis. Lower values indicate stronger statistical significance.

The results are presented in Figure 1, and Figure 2 illustrates a heatmap of biomarker significance across pregnancy trimesters.

### 3.2. Environmental Tobacco Smoke Exposure

Median urinary cotinine concentrations remained below the commonly accepted threshold for passive tobacco smoke exposure throughout pregnancy (1st trimester: 56.7 ± 16.1 ng/mL; 2nd trimester: 47.86 ± 10.5 ng/mL; 3rd trimester: 47.5 ± 10.2 ng/mL), with no significant differences between trimesters.

## 4. Discussion

The present longitudinal study investigated trimester-specific changes in oxidative stress biomarkers and antioxidant status in healthy non-smoking pregnant women. The main finding is a coordinated, marker-specific adaptation of maternal redox homeostasis during physiological pregnancy, characterized by a progressive increase in oxidative damage markers accompanied by selective modulation of antioxidant defenses, while total antioxidant capacity remained stable. These findings are consistent with the concept that pregnancy represents a physiologically regulated pro-oxidant state rather than a pathological oxidative imbalance [17,18,19,39].

A key and clinically relevant observation was the consistent decline in vitamin A across all three trimesters, in contrast to the stable or increasing trends observed for other antioxidants. This pattern is in agreement with previous studies suggesting enhanced maternal–fetal transfer of retinoids, increased metabolic utilization during embryogenesis, and physiological hemodilution due to plasma volume expansion [9,23,38]. Given the essential role of retinoids in embryonic development, cellular differentiation, and immune regulation, this decline most likely reflects increased physiological demand rather than nutritional deficiency. From a clinical perspective, this finding reinforces the importance of maintaining adequate but not excessive vitamin A intake during pregnancy, as both insufficiency and excessive supplementation may have adverse fetal consequences, particularly in early gestation [23].

In contrast, vitamin C remained relatively stable throughout pregnancy, consistent with tight homeostatic regulation of water-soluble antioxidants and efficient recycling mechanisms supporting its roles in collagen synthesis, placental integrity, and immune function [40,41,42].

Lipid-soluble antioxidants exhibited a distinct pattern, with significant increases in α-tocopherol, total vitamin E, and selected carotenoids (xanthophylls and lycopene). These changes are consistent with pregnancy-associated hyperlipidemia and increased lipoprotein-mediated transport, which may enhance systemic antioxidant capacity during periods of elevated metabolic demand [9,42,43]. The non-significant trend observed for β-carotene suggests greater inter-individual variability or reduced robustness after correction for multiple testing.

In parallel, oxidative stress markers demonstrated a clear trimester-dependent increase. Malondialdehyde, conjugated dienes, and protein carbonyls progressively increased, particularly in late pregnancy, reflecting enhanced mitochondrial activity, increased oxygen consumption, and elevated placental metabolic demand [19,24,44]. DNA strand breaks showed only a modest increase between early and late pregnancy without significant changes between adjacent trimesters, suggesting that oxidative stress remains within physiological limits and does not translate into substantial genomic instability under normal conditions [45].

Despite these changes in individual biomarkers, total antioxidant capacity (FRAP) remained stable across pregnancy. This finding indicates that systemic redox buffering capacity is preserved even in the presence of increased oxidative burden. FRAP primarily reflects non-enzymatic reducing capacity and does not capture enzymatic antioxidant systems; however, its stability alongside increased lipid and protein oxidation supports the presence of a compensated physiological pro-oxidant state. Importantly, this stability suggests that antioxidant defenses are functionally reorganized rather than depleted, maintaining overall redox equilibrium throughout gestation.

In this context, tightly regulated redox homeostasis refers to the ability of the maternal organism to maintain overall antioxidant buffering capacity while simultaneously accommodating increased oxidative stress through coordinated and compensatory adjustments in individual antioxidant and pro-oxidant pathways. The observed simultaneous increase in oxidative damage markers and preservation of total antioxidant capacity supports the concept of a dynamic equilibrium rather than uncontrolled oxidative imbalance [17,18,19,39].

Minimal environmental tobacco exposure was confirmed by low urinary cotinine levels, supporting the interpretation that observed redox changes reflect physiological pregnancy adaptations rather than exogenous toxicant exposure [46,47].

Overall, these findings are consistent with previous longitudinal studies demonstrating that physiological pregnancy is associated with increased oxidative stress accompanied by adaptive antioxidant responses [1,24]. The present study extends this knowledge by demonstrating distinct behavior among antioxidant subgroups, particularly the unique decreasing trajectory of vitamin A, which has not been consistently highlighted in previous longitudinal research.

Recent evidence further supports the concept of pregnancy as a controlled redox state influenced by both endogenous and environmental factors. Maternal dietary antioxidant intake has been associated with improved oxidative profiles, whereas environmental exposures such as air pollution may exacerbate systemic oxidative stress during pregnancy [19,25,48]. These findings highlight the multifactorial regulation of redox balance and the interaction between physiological adaptation and external influences.

From a clinical and translational perspective, the observed pattern of redox changes suggests that physiological pregnancy is characterized by adaptive modulation of oxidative stress rather than oxidative damage per se. Although lipid peroxidation and protein oxidation markers increase during gestation, this occurs in parallel with preserved total antioxidant capacity and selective enhancement of lipid-soluble antioxidants. These findings underscore the importance of adequate maternal nutrition, particularly sufficient intake of antioxidant-rich nutrients, while avoiding excessive supplementation, especially of vitamin A.

Although oxidative stress biomarkers show potential as tools for early identification of redox imbalance, their routine clinical application remains limited by methodological variability and lack of standardization. Future multi-marker approaches combining oxidative damage markers with antioxidant profiles may improve their translational utility in prenatal monitoring.

### 4.1. Clinical and Translational Implications

This study suggests that physiological pregnancy is associated with measurable changes in oxidative stress and antioxidant status, reflecting adaptive rather than pathological redox regulation. Disruption of this balance may be involved in pregnancy-related complications, including preeclampsia, gestational diabetes mellitus, and intrauterine growth restriction [4,5,6,7,8,9,10,11].

From a clinical perspective, oxidative stress biomarkers may contribute to the identification of early subclinical redox imbalance. Observed alterations in antioxidant vitamins, particularly the consistent decline in vitamin A and the increase in tocopherols, highlight the importance of adequate maternal nutritional status during pregnancy [20,21,22]. However, these changes should be interpreted within the context of physiological adaptation rather than deficiency or disease.

These findings support preventive strategies including adequate intake of antioxidant-rich nutrients and reduction in exposure to environmental pro-oxidant factors such as air pollution and tobacco smoke [25,26]. At the same time, the routine clinical application of oxidative stress biomarkers remains limited due to methodological variability and lack of standardization, although multi-marker approaches may improve their future utility.

The clinical relevance of oxidative stress biomarkers in pregnancy is summarized in Table 3. This study provides longitudinal data on trimester-specific redox adaptations in healthy pregnancies, which may serve as preliminary reference patterns for physiological oxidative balance. Nevertheless, clinical implementation of oxidative stress biomarkers in prenatal care requires further validation in larger, well-characterized cohorts.

### 4.2. Strengths and Limitations:

Several limitations of this study should be acknowledged. First, the relatively small sample size (n = 31) limits statistical power, particularly for detecting subtle trimester-related changes across multiple biomarkers. No a priori sample size or power calculation was performed; therefore, the findings should be interpreted as exploratory and hypothesis-generating rather than confirmatory.

Second, dietary intake of antioxidants and prenatal vitamin supplementation were not quantitatively assessed. Since circulating levels of vitamins A, C, E, and carotenoids are strongly influenced by diet and supplementation, observed changes may partly reflect inter-individual nutritional variability rather than purely physiological adaptation during pregnancy.

Third, plasma volume expansion during pregnancy was not directly measured. Consequently, changes in absolute biomarker concentrations may be influenced by hemodilution and should be interpreted with this limitation in mind.

Fourth, although all participants were non-smokers and urinary cotinine confirmed minimal environmental tobacco exposure, other lifestyle and environmental factors, such as diet composition and physical activity, were not controlled in detail and may represent residual confounders.

Fifth, enzymatic antioxidant defenses (e.g., superoxide dismutase, glutathione peroxidase, catalase) were not assessed, limiting a complete characterization of the antioxidant system.

Finally, maternal and neonatal outcome data were not systematically collected; therefore, associations between oxidative stress biomarkers and pregnancy outcomes could not be evaluated.

Future studies should include larger cohorts, detailed nutritional assessment, and enzymatic antioxidant markers to provide a more comprehensive characterization of redox homeostasis during pregnancy [7,25,49]. Integration of oxidative stress biomarkers with markers of genomic instability and DNA repair capacity may further enhance understanding of physiological and pathological redox adaptations during gestation [45].

## 5. Conclusions

This longitudinal study demonstrates that physiological pregnancy is characterized by coordinated trimester-specific adaptations in systemic redox balance. Across gestation, a progressive increase in oxidative stress markers was observed, reflecting increased metabolic activity and placental demand.

Despite this increase in oxidative burden, total antioxidant capacity remained stable throughout pregnancy, indicating preserved systemic redox buffering and supporting the concept of a compensated physiological pro-oxidant state rather than oxidative imbalance.

Antioxidant responses were selective rather than uniform. Lipid-soluble antioxidants increased during pregnancy, while vitamin C remained stable. In contrast, vitamin A showed a consistent decline across all trimesters, suggesting a pregnancy-specific adaptation rather than a generalized antioxidant depletion.

Overall, these findings indicate that normal pregnancy is associated with dynamic and tightly regulated redox adaptations that maintain systemic equilibrium despite increasing oxidative demands.

From a translational perspective, the results highlight the importance of balanced maternal nutrition during pregnancy and suggest that oxidative stress biomarkers, although informative for research purposes, require further validation before routine clinical implementation.

## Figures and Tables

**Table 1:** Baseline characteristics of study participants.

Characteristic	Value
Number of participants	31
Maternal age (years)	27.0 ± 4.5
Pre-pregnancy BMI (kg/m^2^)	23.7 ± 2.7
Nulliparous women, n (%)	18 (58.1%)
Multiparous women, n (%)	13 (41.9%)
Non-smokers	100%
Singleton pregnancy	100%

**Table 2:** Overview of analyzed markers and biological samples used.

Biomarker	Sample	Biological Significance
Vitamin A	Plasma	Antioxidant, fetal development
Vitamin C	Serum	Water-soluble antioxidant
α-tocopherol	Plasma	Lipid antioxidant
γ-tocopherol	Plasma	Lipid antioxidant
β-carotene	Plasma	Antioxidant carotenoid
Xanthophylls	Plasma	Antioxidant carotenoids
Lycopene	Plasma	Antioxidant carotenoid
MDA	Plasma	Lipid peroxidation
CDs	Plasma	Early lipid oxidation
PC	Plasma	Protein oxidation
DNA strand breaks	Lymphocytes	Oxidative DNA damage
FRAP	Plasma	Total antioxidant capacity
Cotinine	Urine	Marker of smoking and nicotine use

**Table 3:** Clinical relevance of oxidative stress biomarkers in pregnancy.

Biomarker	Clinical Relevance	Potential Application	Limitations
Malondialdehyde (MDA)	Lipid peroxidation marker	Early indicator of oxidative imbalance; risk assessment	Low specificity; influenced by external factors
Protein carbonyls (PCs)	Protein oxidation marker	Evaluation of systemic oxidative damage	Limited standardization
Markers of DNA damage (e.g., 8-OHdG)	Indicator of oxidative DNA damage	Early detection of cellular stress	Methodological variability
Total antioxidant capacity (TAC)	Overall antioxidant status	Monitoring redox balance	Non-specific marker
Vitamin C	Water-soluble antioxidant	Nutritional assessment	High variability
Vitamin E (tocopherols)	Lipid-soluble antioxidant	Protection against lipid oxidation	Variable plasma levels
Vitamin A (retinol)	Antioxidant and immune function	Nutritional status indicator	Narrow physiological range
FRAP	Marker of total antioxidant capacity	Assessment of antioxidant status and monitoring of dietary or therapeutic interventions	Measures only reducing capacity; influenced by uric acid and vitamin C

## Data Availability

The data presented in this study are available from the corresponding author upon reasonable request.

## Supplementary Materials

The following supporting information can be downloaded at: https://www.mdpi.com/article/10.3390/healthcare14131878/s1.

## Abbreviations

The following abbreviations are used in this manuscript:

ANOVA	analysis of variance
CDs	conjugated dienes
DNA	deoxyribonucleic acid
FRAP	ferric reducing antioxidant power
FDR	False discovery rate
GPx	Glutathione Peroxidase
HPLC	high-performance liquid chromatography
LMM	linear mixed-effects models
MDA	malondialdehyde
PC	protein carbonyl
RNS	reactive nitrogen species
ROS	reactive oxygen species
SD	standard deviation
SOD	superoxide dismutase
