# Supplementary material for: Longitudinal Assessment of Oxidative Stress Biomarkers During Physiological Pregnancy and Their Relevance for Maternal Healthcare
Source: Healthcare (Basel). 2026 Jun 27;14(13):1878. doi: 10.3390/healthcare14131878 (PMC13361728; doi:10.3390/healthcare14131878)
Supplement: Supplementary file 1 [file healthcare-14-01878-s001.zip › Figures and tables.pdf]

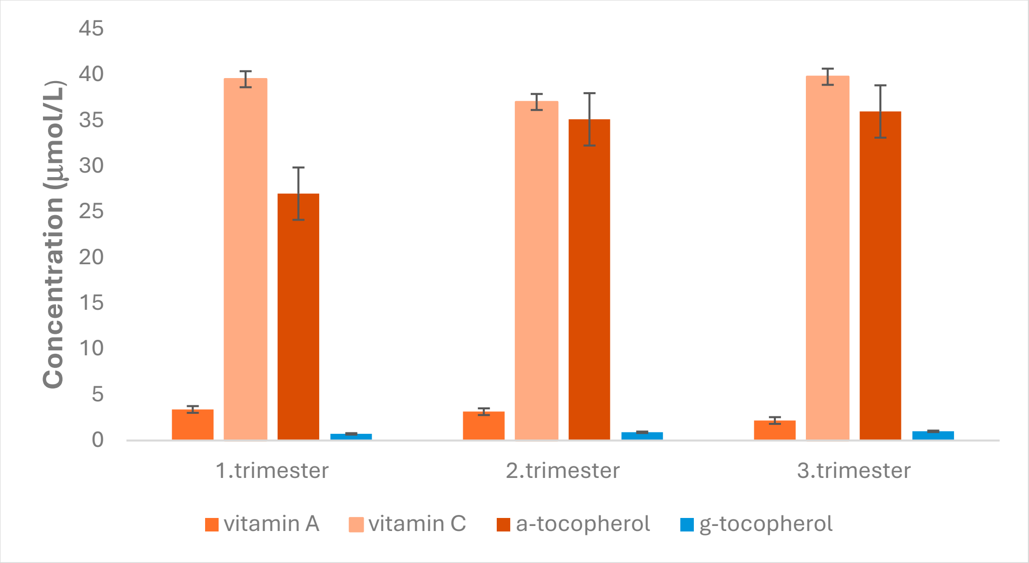

Figure S1. Measured plasmatic concentrations of vitamin A, C, α- and γ-tocopherols in plasma during the 1st, 2nd and 3rd trimesters.

Table S1. Concentrations of carotenoids (β-carotene, xanthophylls, lycopene) in plasma during the 1st, 2nd and 3rd trimesters.

**Carotenoid**	**1st Trimester**	**2nd Trimester**	**3rd Trimester**
β-Carotene	1.46 ± 0.17	1.96 ± 0.23	1.85 ± 0.18
Xanthophylls	0.26 ± 0.03	0.33 ± 0.02	0.41 ± 0.02
Lycopene	0.46 ± 0.03	0.69 ± 0.05	0.64 ± 0.04

Values are presented as means ± standard deviations.

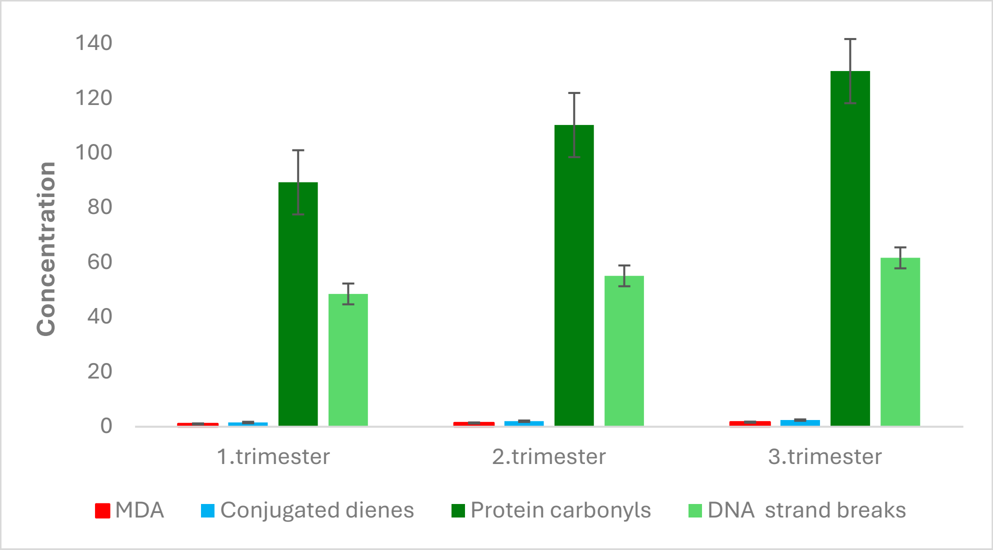

Figure S2. Measured plasmatic concentrations of MDA, conjugated dienes, protein carbonyls (in µmol/L) and DNA strand breaks (in %) during the 1st, 2nd and 3rd trimesters.

Data for constructing Figure S1.

Table S2. Vitamin concentrations (A, C, α-, γ-tocopherol) by trimester.

**Vitamin**	**1st Trimester**	**2nd Trimester**	**3rd Trimester**
A (µmol/L)	3.39 ± 0.17	3.15 ± 0.11	2.81 ± 0.09
C (µmol/L)	39.50 ± 1.67	37.01 ± 2.16	39.77 ± 1.97
α-Tocopherol (µmol/L)	26.99 ± 1.08	35.11 ± 1.48	35.97 ± 1.42
γ-Tocopherol (µmol/L)	0.73 ± 0.05	0.90 ± 0.07	1.00 ± 0.07

Values are presented as means ± SD

Data for constructing Figure S2.

Table S3. Oxidative stress markers (MDA, conjugated dienes, protein carbonyls, DNA strand breaks) by trimester.

Marker	1st Trimester	2nd Trimester	3rd Trimester
MDA (µmol/L)	1.00 ± 0.04	1.35 ± 0.05	1.66 ± 0.05
Conjugated dienes (µmol/L)	1.52 ± 0.05	2.01 ± 0.08	2.42 ± 0.10
Protein carbonyls (µmol/L)	89.3 ± 3.0	110.2 ± 3.6	129.9 ± 4.4
DNA strand breaks (%)	48.5 ± 2.7	55.1 ± 2.9	61.7 ± 3.1

Values are presented as means ± SD.
