# Supplementary material for: Longitudinal Assessment of Oxidative Stress Biomarkers During Physiological Pregnancy and Their Relevance for Maternal Healthcare
Source: Healthcare (Basel). 2026 Jun 27;14(13):1878. doi: 10.3390/healthcare14131878 (PMC13361728; doi:10.3390/healthcare14131878)
Supplement: Supplementary file 1 [file healthcare-14-01878-s001.zip › methods.pdf]

Description of the HPLC methods

Vitamin C in serum

Serum was stabilized by 10% metaphosphoric acid. Level of L-ascorbic acid was measured by high-performance liquid chromatography (HPLC) with diode-array detector using LiChrospher RP 18 reversed-phase column (250x4.0 mm, I.D., 5 μm, Merck, Darmstadt, Germany) as an analytical column and LiChrospher RP 18 (10x4 mm, I.D, 5 μm, Merck, Darmstadt, Germany) as a precolumn. Mobile phase consisted of 3.7 mmol/L potassium dihydrogen phosphate, pH 4.4 (adjusted with H_3PO_4) with flow rate 0.8 mL/min. Injection volume was 25 μL. UV detection was performed at 245 nm [1].

α-tocopherol, γ- tocopherol, retinol, β-carotene, xanthophyll and lycopene in plasma

Lipophilic antioxidants were extracted from plasma using ethanol and hexane containing BHT (butylhydroxytoluene). An internal standard of retinyl palmitate was added to the mixture. Hexane fraction was dried under nitrogen and dissolved in DEA (1,4-dioxane/ethanol/acetonitrile). As an analytical column was applied Nucleosil 120-C18 (250x4.6 mm, I.D., 5 μm, Merck, Darmstadt, Germany) and as a precolumn was used Nucleosil 120-C18 (10x4 mm, I.D, 5 μm, Merck, Darmstadt, Germany). A mixture of acetonitrile/tetrahydrofurane/methanol with butylhydroxytoluene/1% ammonium acetate (67.4:22:6.8:3.8, v/v/v/v) was used as mobile phase with flow rate 1.2 mL/min. Injection volume was 20 μL and column temperature was set at 29°C. UV (450 nm) and fluorescence detectors connected in series. Fluorescence detection was programmed as follows: 0-4 min 330/470 ($\lambda_{exc}/\lambda_{em}$); 4.1-10 min 298/328 ($\lambda_{exc}/\lambda_{em}$) [2-3].

Malondialdehyde in plasma

1,1,3,3 Tetraethoxypropane was used as the MDA standard, and under conditions of acid and heat, condensed with 2-thiobarbituric acid to form a stable adduct (TBA_2-MDA). An alkaline hydrolysis step was then employed, enabling a measure of total plasma MDA to be determined by HPLC. A LiChrospher® RP-18 (125x4 mm, I.D., 5 μm, Merck, Darmstadt, Germany) and a LiChrospher® RP-18 (10x4 mm, I.D., 5 μm, Merck, Darmstadt, Germany) were applied as an analytical column and precolumn, respectively. A mobile phase composed of 50 mmol/L phosphate buffer, pH 6.8, and methanol (60:40, v/v). Isocratic elution was at flow rate 1.5

mL/min. Injection volume was 50 μL. Fluorescence detector was set up at λexc = 532 nm / λem = 553 nm [4].

Protein carbonyls in plasma

The content of carbonyl groups in plasma proteins was determined spectrophotometrically. The method is based on the observation that reactive products of oxygen metabolism can attack amino acids to form carbonyl groups. The carbonyl groups react with dinitrophenylhydrazine to form dinitrophenylhydrazone detected at 365 nm [5].

Products of oxidative DNA damage in isolated lymphocytes

The alkaline comet assay was used to detect DNA breaks, oxidized purines, and oxidized pyrimidines in isolated lymphocytes [6-8]. The Comet assay method is based on the ability of single-stranded DNA to unwind from the DNA helix. The negatively charged ends of the DNA travel in an electrophoretic field to the positively charged pole, forming a tail extending from the nucleus. The intensity of fluorescence in the comet tail is a function of DNA damage, i.e. the relative amount of DNA in the tail expresses the amount of DNA breaks. The preparations were evaluated with an Olympus BX40 fluorescence microscope with filters in the UV region. The degree of damage was assessed according to the length of the tail and the fluorescence intensity in the head and tail visually. Four degrees of damage were determined during the visual assessment. DNA damage is expressed in arbitrary units in the interval from 0 to 400, i.e. maximum DNA damage has a value of 400.

Conjugated fatty acid dienes in plasma

The value of the first lipid peroxidation product was determined spectrophotometrically [9]. Conjugated dienes in the heptane phase were measured at 233 nm.

1. Richard, M. J.; Guiraud, P.; Meo, J.; Favier, A.: High-performance liquid chromatographic separation of malondialdehyde-thiobarbituric acid adduct in biological materials (plasma and human cells) using a commercially available reagent. *J Chromatogr* **1992,** *577*, 9-18.
2. Cerhata, D.; Baureová, A., Ginter, E.: Determination of ascorbic acid in blood serum using high-performance liquid chromatography and its correlation with spectrophotometric (colorimetric) determination. *Ceska Slov. Farm.* **1994**, *43*, 166–168.

3. Lee, B.L.; Chua, S.C.;, Ong, H.Z., Ong, H.Y. High performance liquid chromatographic methods for routine determination of vitamins A and E, and β- carotene in plasma. *J. Chromatogr*. **1992**, *58*1, 41- 43.
4. Talwar, D.; Ha, T.K.K.; Cooney, J.; Brownlee, C.; O´Reilly, D.S. A routine method for the simultaneous measurement of retinol, α-tocopherol and five carotenoids in human plasma by reverse phase HPLC. *Clin. Chim. Acta* **1998**, *270*, 85-100.
5. Levine, R.L.; Garland, D.; Oliver, C.N.; Amici, A.; Climent, I.; Lenz, A.G.; Ahn, B.W.; Shaltiel, S.; Stadtman, E. R. Determination of carbonyl content in oxidatively modified proteins. *Methods in Enzymol*. **1990**, *186*, 464-478.
6. Recknagel, R.; Glende, E.A. Spectrophotometric detection of lipid conjugated dienes. *Methods Enzymol.* **1984**, *105*, 331-337.
7. Collins, A. R., Dušinská, M., Gedik, C. M., Stetina, R.: Oxidative Damage to DNA: Do have a reliable biomarker? *Environ. Health Perspect*., **1996,** *104*, 465- 469.
8. Collins, A., Dušinská, M., Franklin, M., Somorovská, M., Petrovská, H., Duthie, S., Panayiotides, M., Rašlová, K., Vaughan, N.: Comet assay in human biomonitoring studies, reliability validation and application. *Environ. Mol. Mutag*., **1997**, *30,* 139-146.
9. Dušinská, M., Collins, A.: Detection of oxidised purines and UV-induced photoproducts in DNA of single cells, by inclusion of lesion-specific enzymes in the comet assay. *ATLA,* **1999,** *24*, 405-411.
